# Editorial: Fontan-associated liver disease: current status and future directions

**DOI:** 10.3389/fmed.2025.1575638

**Published:** 2025-03-25

**Authors:** Ornella Milanesi, Oktay Tutarel, Annalisa Angelini

**Affiliations:** ^1^Department of Women and Children's Health University of Padova, Padova, Italy; ^2^Deutsches Herzzentrum der Charité, Department of Congenital Heart Disease – Pediatric Cardiology, Berlin, Germany; ^3^Charité - Universitätsmedizin Berlin, Corporate Member of Freie Universität Berlin and Humboldt-Universität zu Berlin, Berlin, Germany; ^4^Pathology of Cardiac Transplantation and Regenerative Medicine Unit, Department of Cardiac Thoracic and Vascular Science and Public Health- University of Padova, Padova, Italy

**Keywords:** Fontan principle, liver cirrhosis, hepatocellualr carcinoma, liver fibrosis, protein losing enteropathy (PLE)

Fontan Associated Liver Disease (FALD) is an emerging clinical syndrome, which is the unavoidable consequence of the specific hemodynamic condition of the Fontan procedure. In 1971, Francis Fontan published the result of his visionary procedure designed to treat patients with tricuspid atresia, which consisted in the anastomosis of the superior vena cava to the right pulmonary artery and of the inferior vena cava to the left pulmonary artery by means of the interposition of a homograft between the right atrium and the left pulmonary artery (1). He demonstrated that the right ventricle is a dispensable chamber. Since then, thousands of patients with anatomic or functional single ventricle have been treated, and are now surviving and reaching adulthood due to the Fontan principle, along with its numerous surgical modifications to enhance the hemodynamics of the circuit (2, 3).

Over time, congenital cardiologists monitoring these patients have come to realize that the systemic venous pressure overload required to drive pulmonary circulation has significant consequences on abdominal organs, particularly the liver. The term Fontan Associated Liver Disease (FALD) was subsequently coined to describe the changes caused by the systemic venous hypertension and congestion over time in the livers of the Fontan patients, leading to progressive fibrosis, cirrhosis, and even an increased risk of hepatocellular carcinoma (4).

Although this new nosographic entity has been recognized in clinical practice, we still lack standardized diagnostic tools to determine the point at which a Fontan patient should be diagnosed with FALD. Additionally, reliable methods to monitor the progression of liver disease over time and identify when a heart transplant is necessary to prevent irreversible liver dysfunction are not yet available. This important entity is the topic of this collection. Its relevance even more evident by what has been highlighted in the recent publication (5) of the data of the EUROFONTAN registry, which demonstrate that in almost 30% of the European surgical centers participating in the registry assessing the liver functionality is not included in the follow-up of Fontan patients.

Rising the awareness of the Pediatric Cardiology community on the necessity of a well-organized multidisciplinary team, is well underlined in Tsuchihashi et al. article.

As emphasized in Tsuchihashi et al.'s contribution, the chronic pressure overload of the liver in Fontan patients causes histological remodeling. Therefore, liver biopsy should be still considered the gold standard for the diagnosis. In fact, non-invasive tools are debated as valid alternative to biopsy. However, in these patients the lack of an established histologic scoring system for FALD diminishes the relevance of histologic assessment for diagnosing FALD. The explanation is that conventional histologic criteria for liver fibrosis, including the Metavir and Ishak scores and the method of Scheuer, have been applied only for the chronic liver disease associated with HCV and HBV, focusing on liver fibrosis in the portal region. These conventional methods are considered inappropriate for assessing the unique histologic features of FALD, which include sinusoidal fibrosis and dilation. Modifications to existing scoring systems, as well as the newly proposed Congestive Hepatic Fibrosis Score (CHFS) (6), have been found to be suitable for this patient population.

Regarding the feasibility of the liver biopsy in Fontan patients, Mandilaras et al. contribution offers the readers the precise procedural information on the technical aspects of the liver biopsy in Fontan patients. The surgical anatomy of the Fontan circulation allows an easy access to the liver through a trans-jugular approach and contributes to the safety of the procedure. The authors suggest including liver biopsies in the routine follow-up cardiac catheterization, which is recommended for all Fontan patients. An important aspect which has been underlined by the authors is that as the biopsy site is intravascular, the risk of external bleeding or hematoma is significantly reduced despite the high intrahepatic pressures and the usually impaired coagulation profile in these patients.

Another important contribution to the understanding of the scenario in which patients with Fontan circulation live in Europe nowadays is contained in the Meyer et al. article. They report on the results of a German nationwide survey carried out to investigate the use of transient liver elastography in the routine follow-up of those patients.

Transient liver elastography is a non-invasive method to evaluate the liver and spleen stiffness. Although it correlates with the engorgement of the liver and not directedly with the severity of the fibrosis/cirrhosis (7, 8) it is a non-invasive method which can give important information on the pressure in the Fontan circuit, and the presence of functional and or anatomical problems which could be potentially treated, reducing the hemodynamic burden to the liver and improving the outcome of these patients. The survey demonstrated that 40% of the German Centers do not perform at any time surveillance of the liver stiffness by means of transient elastography and furthermore that it is proposed on a routine basis only in 13% of the Centers.

An interesting data on transient liver elastography, is presented by Braun et al.. In subjects with normal biventricular circulation, in whom liver elastography is performed to evaluate the presence of fibrosis consequent to liver disease, the test must be performed after at least 6 h fasting and the result is affected by inspiration and expiration (9). The authors performed transient elastography in 25 Fontan patients and 50 healthy volunteers, after at least 6 h of fasting and 15, 30, 45, 60, 90, 120, 150, and 180 min after ingestion of 500 ml of chocolate drink, and both during in and expiration. They demonstrated that the liver stiffness in those patients is not affected by any physiologic condition but by the pressure in the Fontan circuit.

This information is another important adjunct to what is known on this Research Topic.

In conclusion, we believe that this Research Topic provides a window of opportunity to improve the still insufficient awareness of the emerging burden of FALD in the Pediatric Cardiology and Cardiac Surgery community in Europe, and proposes efficient follow-up methodologies, with an emphasis on the requirement of a multidisciplinary effort.
